# Case Report: SPECT-CT-guided minimally invasive transverse process resection for Bertolotti syndrome

**DOI:** 10.3389/fsurg.2025.1733483

**Published:** 2026-01-20

**Authors:** Samantha E. Spellicy, Ellen O'Callaghan, Michael Patetta, Dennis A. Turner, Muhammad M. Abd-El-Barr

**Affiliations:** 1 Department of Neurosurgery, Duke University Hospital, Durham, NC, United States; 2 Department of Orthopaedic Surgery, Duke University Hospital, Durham, NC, United States

**Keywords:** Bertolotti syndrome, lumbar surgery, lumbosacral transitional vertebrae (LSTV), minimally invasive spine surgery, SPECT-CT

## Abstract

Lumbosacral transitional vertebrae (LSTV) are a common congenital anomaly that often manifests as chronic low back or radicular pain, a condition clinically referred to as Bertolotti syndrome. One specific cause of Bertolotti syndrome is pseudoarticulation of the L5 transverse process with the sacrum or ilium due to LSTV. Although conventional magnetic resonance imaging (MRI) and computed tomography can identify structural changes, they provide limited functional information regarding sites of active arthropathy. Single-photon emission computed tomography (SPECT-CT) enables the localization of metabolically active pseudoarthrotic joints, thereby improving patient selection and surgical planning. We present the case of a 52-year-old woman with Bertolotti syndrome who presented with severe chronic axial back pain and left-sided pain radiating along portions of the L5 and S1 dermatomes. MRI revealed no significant compression of the neural elements but did demonstrate an incidental Tarlov cyst at S1, measuring 5.7 mm × 5.7 mm. SPECT-CT demonstrated localized, abnormal uptake between an anomalous left L5 transverse process and the sacrum. The patient underwent minimally invasive, image-guided removal of the left L5 transverse process, isolating L5 vertebral motion from the iliac crest. She was discharged on postoperative day 1 with significant improvement in her pain and radiculopathy. At 6-week, 3-, and 6-month follow-up, she reported near-complete resolution of presurgical radicular pain, functional restoration, and a return to normal activities. This case highlights the utility of SPECT-CT in evaluating Bertolotti syndrome. Functional imaging enabled precise structural localization of the pain generator, while targeted minimally invasive resection provided durable symptom relief.

## 1 Introduction

Lumbosacral transitional vertebrae (LSTV) are a relatively common spinal anomaly affecting between 4% and 35% of the general population (1, 2). A classification system for LSTVs based on morphological and clinical characteristics was first introduced in 1984 by Castellvi. This system defines LSTV as any articulation of the L5 vertebra with the sacrum or ilium, including abnormal enlargement or sacralization of an aberrant transverse process (TP) (3, 4). Explicitly, type I is characterized by an enlarged dysplastic TP (at least 19 mm), type II involves pseudoarticulation of the TP and sacrum with incomplete lumbarization and pseudoarthrosis, type III includes fusion of the TP with the sacrum and incomplete lumbarization but complete fusion, and type IV represents a mix of type II and type III changes on each side. Subtypes of types I, II, and III are designated “a” for unilateral involvement and “b” for bilateral involvement (3).

Since the introduction of the Castellvi classification, additional classification schemes have been proposed that further subdivide these descriptions, particularly to help separate surgical treatment approaches (5, 6). Bertolotti syndrome refers to a clinical diagnosis in patients who present with lumbar axial pain or radicular pain that can be directly attributed to LSTV identified on computed tomography (CT) or plain X-ray (7) following extensive clinical evaluation (1, 4–6, 8–11). The cause of this associated pain and the optimal treatment options remain debated topics (11). In a subset of patients with LSTV, pain is thought to arise from abnormal pseudoarticulation between the LSTV and the sacrum and/or ilium. Recent updates for Bertolotti syndrome outline a stepwise approach, beginning with conservative management (4–6), including ipsilateral or contralateral transforaminal epidural steroid injections at either L4–L5 or L5–S1 (12, 13), interlaminar epidural injections at L4–L5 (13), lumbar facet medial branch blocks (13), and sacroiliac joint (SI) injections (13). These conservative measures are traditionally followed by eventual surgical intervention, including transverse processectomy, decompression and fusion (4–6), and dorsal ramus lateral branch radiofrequency neurotomy (13). Reviews of these surgical approaches have found that the success rates of these surgical techniques, defined as postoperative pain reduction, range from 0% to 77%, based on data from both multiple case series (5, 10, 14–16) and case reports (9, 15, 17–23).

Appropriate patient selection, accurate pathological localization, thorough preoperative counseling, and selection of either minimally invasive or lumbar fusion-based surgical approaches are crucial for improving postoperative outcomes (4–6, 8, 16). When considering patient selection and operative approaches, current imaging modalities, such as traditional magnetic resonance imaging (MRI) and three-dimensional MRI, are valuable for identifying targetable thecal or nerve root compression (1). Computed tomography (CT) is also instructive, as it can help identify bony points of articulation of the LSTV and signs of abnormal bone response to pseudoarticulation. These modalities, however, lack dynamic information, such as areas of active metabolism or inflammation, which may help identify arthritic sites that could be contributing to a patient's symptoms. Single-photon emission computed tomography (SPECT-CT) bridges this gap by enabling the identification of pseudoarthrotic sites through abnormal radiotracer uptake (24, 25). This imaging modality is invaluable for preoperative optimization, as it enables direct visualization of targetable pathology. Furthermore, in patients with multiple complicated pathologies, such as nerve root compression at multiple lumbar levels on MRI or multiple potential pseudoarticulations, SPECT-CT can help identify sites of active inflammation to refine the surgical approach and plan interventions with the highest likelihood of symptom relief. Our group has also demonstrated the prognostic utility of SPECT-CT in predicting patient outcomes. In patients who underwent lumbar interbody fusion for chronic low back pain, increased SPECT-CT uptake within the disc space was significantly associated with decreased pain scores at 1 year postoperatively (26). Interestingly, this correlation was location-specific, as increased facet SPECT-CT uptake was not significantly correlated with improved pain scores. Because of this location-specific prognostic utility, we aim to build upon the work of previous groups, which have demonstrated increased abnormal SPECT-CT uptake in patients with Bertolotti syndrome (11, 24, 25), and better elucidate the role of SPECT-CT in patient selection, surgical approach, and postoperative pain outcomes.

Here, we present an operative case of a 52-year-old woman with unilateral Bertolotti syndrome type 2A (5, 6) who presented with chronic lumbosacral pain and radiculitis radiating to her bilateral gluteal regions, without any sensory or motor deficits. Abnormal radiotracer uptake was observed at the site of contact between the sacrum and an anomalous left L5 transverse process. MRI also identified a Tarlov cyst at the S1 level in the patient, which measured 5.7 mm in radius on sagittal and coronal views, corresponding to an estimated volume of 0.776 cm^3^. Based on the SPECT-CT results, as well as the patient's history and symptoms, the decision was made to address the left L5 TP alone, as suggested by the grading classifications, rather than pursue a more extensive lumbar fusion (5, 6). As anatomical constraints associated with LSTV, such as the position of the iliac crest, can limit traditional surgical corridors, a minimally invasive approach using a tubular system (Globus Medical, Audubon, PA) with image guidance (BrainLab, Munich, Germany) was selected for this case (15, 16, 18, 21, 23). This minimally invasive (MIS) approach provided direct access to the anomalous left L5 TP process for resection while minimizing surgical complications and decreasing postoperative recovery time.

## 2 Case description

A 52-year-old woman with a past medical history significant for migraines, fibromyalgia, and ulnar neuropathy presented to the neurosurgery spine clinic in 2023 with a complex history of lumbosacral and right lower extremity pain. Prior to her neurosurgical presentation, she had undergone right ankle tendon repair in 2019, which was complicated by cellulitis, severe residual pain, episodic swelling, and hypertrophic scarring. In 2020, she was diagnosed with complex regional pain syndrome type 1 (CRPS1) involving the right ankle. During this period, she also experienced lumbosacral back pain that was refractory to duloxetine and pregabalin, both of which were discontinued due to ineffectiveness and mental fog. In 2021, the patient underwent four sympathetic blocks at the right L3 over a 3-month period for the management of CRPS type 1 under the anesthesia pain service (APS). As the blocks provided some pain relief, she was recommended for a dorsal root ganglion (DRG) stimulator trial in 2022, with leads placed at the right S1 foramen and right L5 foramen. Unfortunately, the procedure was complicated by cerebral spinal fluid (CSF) leak and positional headache, which necessitated device removal and placement of a blood patch on postoperative day 1. Due to the rapid trial termination, the therapeutic efficacy of the DRG stimulator remained unknown.

### 2.1 Diagnostic assessment

**Figure 1:**
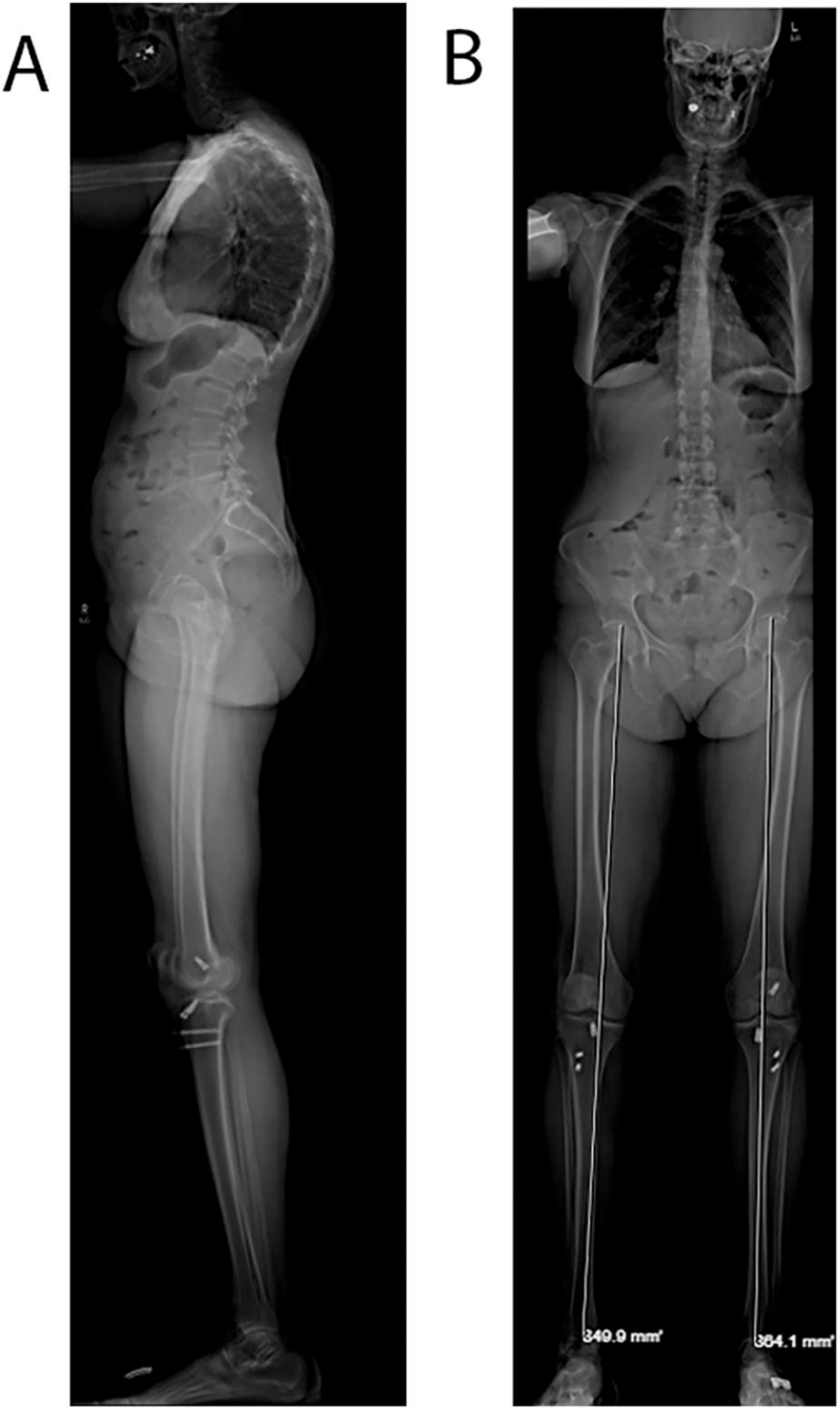
EOS spinal alignment imaging. Upright lateral **(A)** and anterior–posterior **(B)** EOS images demonstrating the patient's normal sagittal alignment with slightly increased thoracic kyphosis.

**Figure 2:**
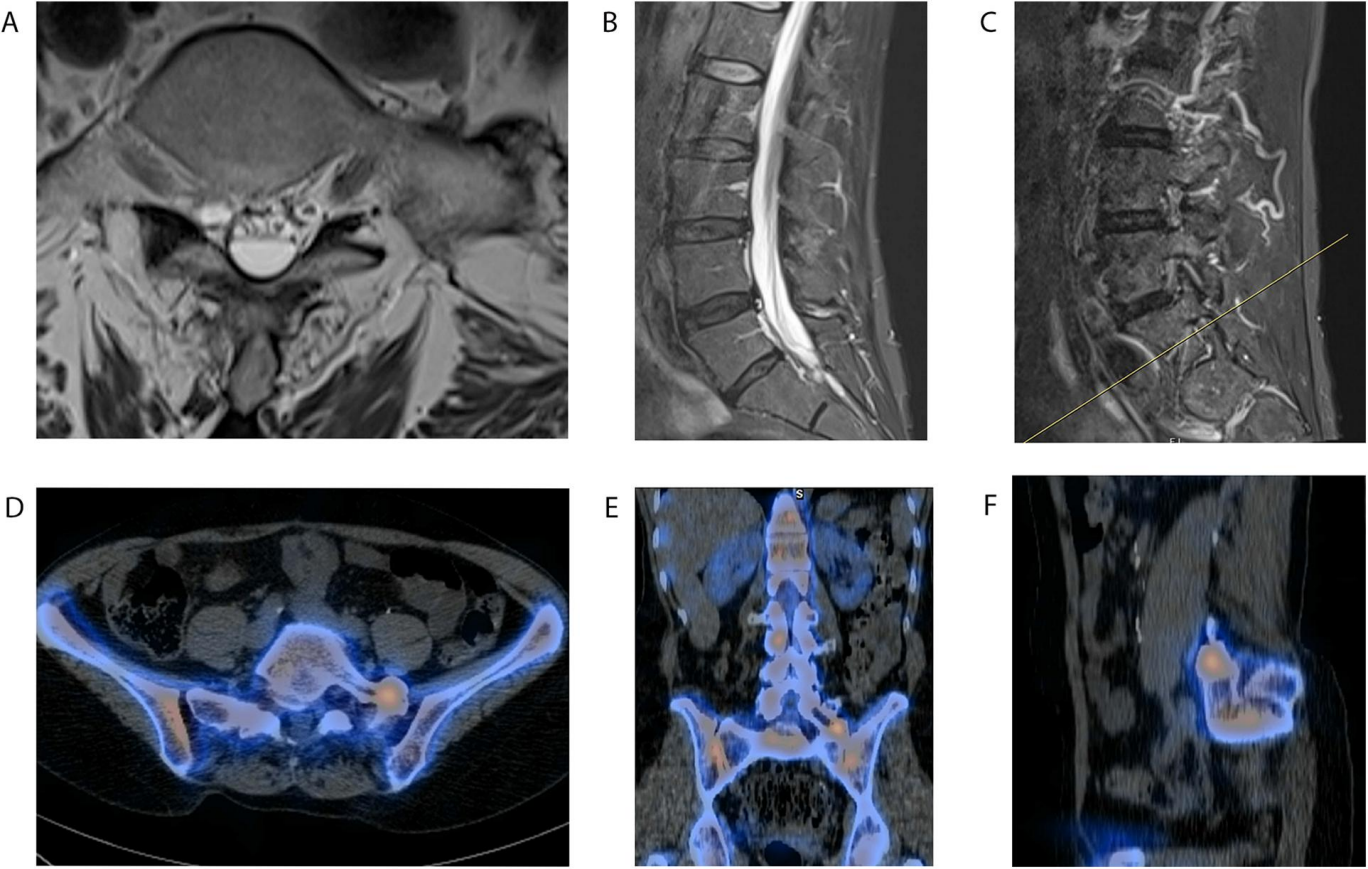
Preoperative lumbar magnetic resonance imaging (MRI) and CT-SPECT**.** lumbar T2-weighted axial **(A)**, midline sagittal **(B)**, and contrast-enhanced left-sided sagittal **(C)** MRI images demonstrating no obvious thecal compression above the LSTV and no obvious neuroforaminal stenosis. An incidental T2-intense cystic-appearing lesion, likely a Tarlov cyst, is identified at the LSTV and is most evident on axial imaging **(A)**, where it anteriorly displaces the cauda equina nerve roots. SPECT-CT identifying unilateral abnormal radiotracer uptake at the left L5 TP near the articulation with the sacrum on axial **(D)**, coronal **(E)**, and sagittal **(F)** views . This abnormal uptake signifies a higher metabolic activity at the site of the left TP than would be expected. Abnormal uptake is not seen on the contralateral right side at this level.

Throughout her neurosurgical course, the patient's description of pain evolved. At her initial neurosurgical presentation in the spring of 2023, she described her pain as right-worse-than-left lumbosacral back pain radiating across the belt line into the right buttock. She also experienced lower extremity pain localized to the right ankle. She stated that her low back pain was exacerbated by exercise, lifting, lying down, rest, sitting, standing, and walking and was improved primarily through activity modification. She had a PROMIS Physical Function T-score of 41 (consistent with mild dysfunction). On physical examination, she demonstrated right-greater-than-left sacroiliac joint (SI) pain that was exacerbated at extremes of motion, without motor or sensory deficits. Her lumbosacral and radicular back pain was located along the L5 and S1 dermatomes, with symptoms more pronounced on the left than the right. She also exhibited tenderness to palpation of the paraspinal and bilateral SI joints. Electro-optical system (EOS) spinal imaging demonstrated overall good alignment (Figure 1). MRI was acquired for further workup and revealed a right-sided sacral Tarlov cyst at the S1 level measuring 5.7 mm × 5.7 mm, which was stable compared with prior MRI from 2022 (Figures 2A–C). SPECT-CT imaging was acquired (Figures 2D–F) and demonstrated LSTV with focal radiotracer uptake at the site of contact between an anomalous left L5 TP and the sacrum, as well as subtle uptake in the bilateral SI joints. Due to the patient's tenderness with palpation of the bilateral SI joints and pain in the right SI joint at extremes of motion, she was first referred for right-sided CT-guided SI joint injection. Two months following the SI joint injection, and 4 months after her first neurosurgical presentation, the patient stated persistent achy lumbosacral pain, with new onset of left buttock pain. She also described pain involving both lower extremities; however, it was difficult for her to describe the character and localization due to confounding chronic pain in her bilateral knees (for which she had prior surgeries) and right ankle CRPS type 1. She denied radicular pain radiating down her bilateral lower extremities (BLEs), was full strength with no sensory deficits, and reported no urinary or fecal incontinence. Her PROMIS Physical Functional T-Score following right SI joint injection was 42, practically unchanged from the score of 41 at her first neurosurgical presentation.

After consideration of the L5/S1 transitional anatomy and focal radiotracer uptake at the articulation between the left L5 TP and the sacrum on SPECT-CT, the neurosurgery team raised concern for Bertolotti syndrome (21, 22). While Bertolotti syndrome can be diagnosed using X-ray imaging (7), SPECT-CT proved invaluable in refining the diagnosis in this case, as her chronic joint pain history, fibromyalgia, and CRPS complicated her workup. After recognition of this pseudoarthrosis site with focal radiotracer uptake on SPECT-CT, she was recommended for facet injections at her L5/S1 transitional anatomy. She then underwent CT-guided injections on the left into the pseudoarthrosis L5/S1 site and on the right through an L5–S1 pars defect. A posterior oblique approach was used for both procedures, with the intra-articular/periarticular location confirmed by contrast injection. At the next follow-up, approximately 2 months later, the patient stated that the injections did “decrease her pain significantly, more so on the left side,” but it did not resolve all her lumbosacral and gluteal pain. She did find that relief of her left gluteal pain allowed her to be more active; therefore, a second round of CT-guided injections using the same approach at the bilateral location was recommended. At her next follow-up 3 months later, the patient reported that the second injection provided less benefit than the first regarding her lumbosacral axial pain, providing only 50%–60% pain relief for a “short” duration. Throughout this workup, she remained full strength with no sensory deficits.

Approximately 1 year later, the patient returned to the neurosurgery spine clinic with worsening lumbosacral axial back pain, now predominantly left-sided, with pain radiating to the left leg, hip, and buttock along the L5 and S1 dermatomes. She described the pain as constant, exacerbated by standing, walking, and sitting, and significantly impacting her daily life activities. She reported adjusting her activities to cope with the pain and found it challenging to maintain her usual routine. No new weakness or sensory changes were identified upon examination. As conservative measures, including SI joint injections and physical therapy, had provided no relief, and an L5/S1 transitional anatomy facet injection had provided only temporary relief, surgical intervention was considered. Given the description and location of her pain, coupled with imaging findings on SPECT-CT showing focal radiotracer uptake at the L5 transverse process articulation with the sacrum, the decision was made to proceed with minimally invasive resection and decompression of the L5 left transverse process.

### 2.2 Therapeutic intervention

**Figure 3:**
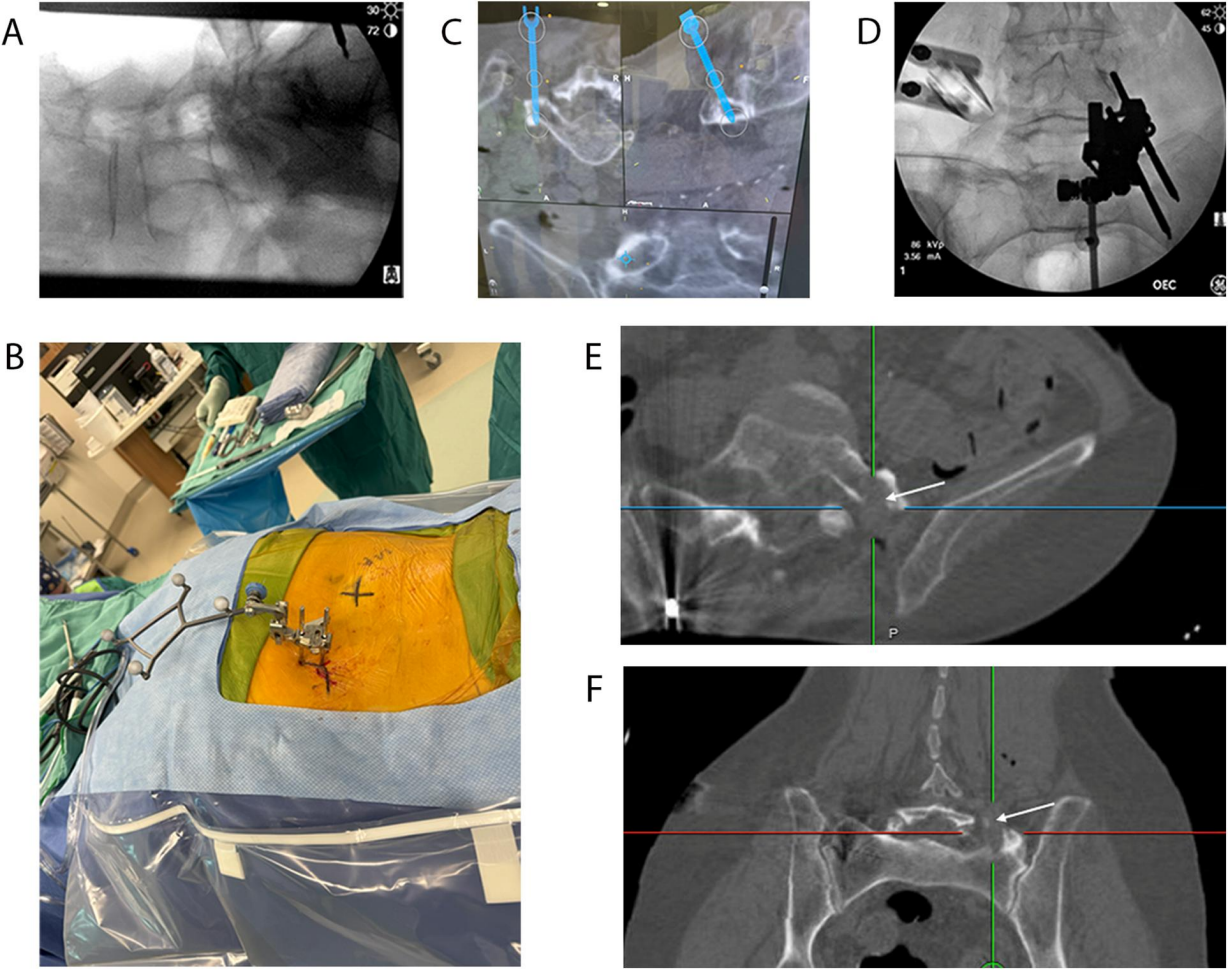
Intraoperative localization and navigation. Lateral intraoperative X-ray **(A)** indicating the position of the brain lab fiducial marker in the superior iliac spine (ASIS). The intraoperative image demonstrates the location of the BrainLab fiducial marker in the ASIS and the left-sided skin incision site (marked with a black “X”). Intraoperative brain lab navigation demonstrating live axial, sagittal, and coronal views with the trajectory **(C)** used to dock the spine tube system directly onto the left anomalous L5 TP. Anterior–posterior intraoperative X-ray **(D)** demonstrating the BrainLab fiducial positioned in the ASIS and the spine tube system after insertion. Intraoperative CT obtained after left anomalous TP resection demonstrating completed processsectomy and removal of pseudoarthrotic site in the axial **(E)** and coronal **(F)** planes.

The patient was taken to the operating room, placed in the prone position, and prepped. A fiducial marker was placed in the right posterior superior iliac spine for localization (BrainLab), with position confirmed on intraoperative X-ray (Figure 3A). CT imaging was performed with a subsequent planned trajectory toward the left pseudojoint to be completed using the spine tube system. Measurements obtained from the preoperative CT, in addition to intraoperative tracking (BrainLab), were used to determine the superficial entry point for a left-sided incision (Figure 3B). The underlying fascia was incised using electrocautery. Tracking (BrainLab) was utilized to guide dissection from the skin incision down to the anomalous left L5 transverse process (Figure 3C). Once the position was confirmed using intraoperative tracking, sequential dilators were utilized through the spine tube system, which exposed the left L5 TP. X-ray fluoroscopy was used to verify the L5 level (Figure 3D). The microscope was then positioned to provide a full view of the anomalous transverse process through the spine tube system. A 5-mm diamond drill was then used to resect the left L5 TP and to completely disconnect the L5 vertebral body from the iliac crest. Soft tissue was encountered anteriorly, superiorly, and laterally (toward the iliac crest). Inferiorly, the sacral ala was used to direct the lower aspect of the drilling. A thin wall of bone over the L5 nerve root was left medially. A nerve root stimulator was inserted medially and stimulated to 2–3 mA, showing subsequent L5 contractions in the left leg, confirming the medial extent of the resection (20). As the surgical goals were accomplished with sufficient resection of the TP, the thin medial wall of bone was left intact, as further intervention would have increased the risk of injury to the exiting L5 nerve. Intraoperative CT imaging confirmed resection of the anomalous joint and disconnection between L5 and the sacral ala and iliac crest (Figures 3E,F).

The patient was deemed safe for discharge on postoperative day 1, with independent ambulation, tolerance of a normal diet, and good pain control.

## 3 Discussion

Often, in cases of complex pain with multiple potential etiologies of the patient's symptoms, such as in the case presented here, it is difficult to accurately identify an area of appropriate surgical intervention. This difficulty is further complicated in Bertolotti syndrome, in which the root cause of the associated pain appears to be mechanical, limiting the normal motion of the L5 vertebral body (4–6, 27). While some groups have postulated that the pain is from central or peripheral compression of elements above the level of the LSTV or extraforaminal nerve compression and compromise, others propose that symptoms result from bony contact or pseudoarticulation between an aberrant L5 TP and the sacrum, or from contralateral facet degeneration secondary to abnormal ipsilateral joint fusion (5, 6, 10, 27). Joint and epidural injections can assist with pain localization and surgical planning and are included in the stepwise evaluation of Bertolotti syndrome. However, outcomes from these interventions may be inconclusive, with pain relief often limited or temporary. SPECT-CT serves as a useful modality in conjunction with an established stepwise workup of Bertolotti's syndrome for identifying pathological and targetable sites (24, 25).

**Figure 4:**
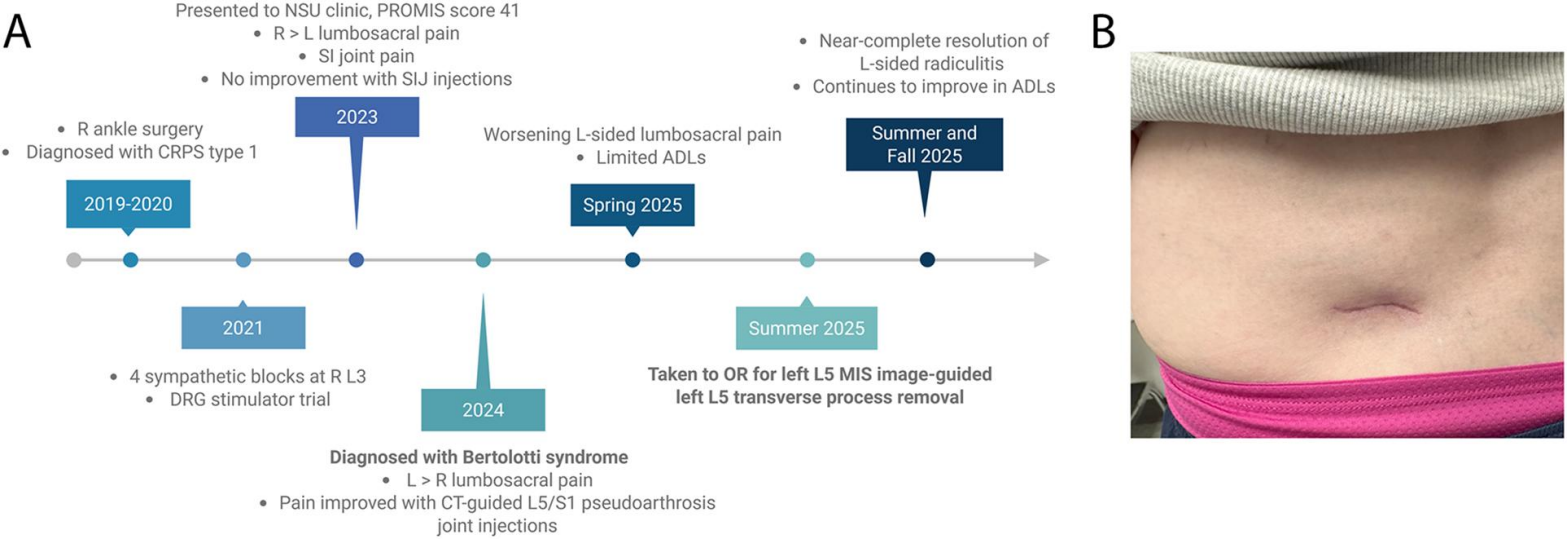
Clinal course of patient's presentation and postoperative outcomes. Timeline of patient's clinical course, including CPRS type 1, failure of SI injections, worsening left-sided pain, responsive L5/S1 pseudoarthrosis site injections, operative intervention, and postoperative course. Created in https://BioRender.com. **(A)**. Left flank MIS incision site at the 6-week follow-up visit, measuring less than 2 inches and demonstrating expected healing **(B)**.

In this presented case, the patient experienced initially right-sided and then left-sided radicular symptoms, with no relief following SI joint injections, as well as an underlying transitional anomalous anatomy and an incidental Tarlov cyst. Furthermore, the patient's history was complicated by a history of complex regional pain syndrome of the right lower extremity and a failed DRG stimulator trial (Figure 4A).

The decision was made to proceed with a left-sided L5 transverse processsectomy rather than other potential surgical interventions. Specifically, potential alternatives included central and lateral recess decompression for moderate stenosis related to the Tarlov cyst or fusion of the transitional anatomy. Without the localization provided by SPECT-CT, an alternative surgical approach might have been pursued, resulting in unsuccessful postoperative pain relief.

At the most recent follow-up, the patient continues to report complete resolution of her preoperative radicular pain. We believe that SPECT-CT was instrumental in achieving symptom resolution in this case, given her prolonged and complex pain history involving multiple etiologies. While the patient continues to experience knee, hamstring, and tendonitis-like pain, she affirmed that the resolution of her radicular pain has significantly improved her mobility and ability to perform activities of daily living. We attribute the success of this case to a targeted SPECT-CT-guided MIS approach.

## 4 Patient perspective

The patient returned to the clinic for scheduled follow-up at 6 weeks and 3 months (virtually) following the intervention. At her 6-week postoperative visit, she reported near-complete resolution of her back and radicular pain. Her incision was well healed (Figure 4B), and she demonstrated full (5/5) strength and intact sensation throughout the lower extremities. She stated that she “would do it [surgery] again in a heartbeat.” She was cleared to return to work without restrictions and approved to undergo knee surgery 4 months after the neurosurgical intervention for her chronic knee pain.

At her virtual 3-month postoperative appointment, the patient reported new-onset back pain that differed in character and intensity from her preoperative pain. She described the pain as lower and more central than her prior pain, localized toward her tailbone. She rated the pain as a 1–2 out of 10 in severity. She denied any radiating pain through her hips or either leg, which is in contrast to the severe, debilitating pain she experienced prior to neurosurgical intervention. Of note, she had been wearing a boot for approximately 4 weeks for severe tendonitis and hamstring pain and had been undergoing physical therapy. She continues to undergo physical therapy weekly for her leg pain and is “hopeful that her upcoming knee surgery will help address her other pains.” She is scheduled for an in-person follow-up visit following her knee surgery to further monitor her postoperative recovery.

## Data Availability

The original contributions presented in the study are included in the article/Supplementary Material, further inquiries can be directed to the corresponding author.
